# CUL1 promotes breast cancer metastasis through regulating EZH2-induced the autocrine expression of the cytokines CXCL8 and IL11

**DOI:** 10.1038/s41419-018-1258-6

**Published:** 2018-12-18

**Authors:** Ye-Fei Huang, Zhe Zhang, Meng Zhang, Yan-Su Chen, Jun Song, Ping-Fu Hou, Hong-Mei Yong, Jun-Nian Zheng, Jin Bai

**Affiliations:** 1 Cancer Institute, Xuzhou Medical University, 221002 Xuzhou, Jiangsu Province China; 2 School of Public Health, Xuzhou Medical University, 221002 Xuzhou, Jiangsu Province China; 3 Center of Clinical Oncology, Affiliated Hospital of Xuzhou Medical University, 221002 Xuzhou, Jiangsu Province China; 4 Department of General Surgery, Affiliated Hospital of Xuzhou Medical University, 221002 Xuzhou, Jiangsu Province China; 5 Jiangsu Center for the Collaboration and Innovation of Cancer Biotherapy, Xuzhou Medical University, 221002 Xuzhou, Jiangsu Province China; 6 Department of Medical Oncology, Huai’an Hospital to Xuzhou Medical University, 223001 Huai’an, Jiangsu Province China

## Abstract

CUL1 is an essential component of SCF (SKP1-CUL1-F-box protein) E3 ubiquitin ligase complex. Our previous study has showed that CUL1 is positively associated with poor overall and disease-specific survival of breast cancer patients. Here, we further explored its roles in breast cancer metastasis. Our data showed that CUL1 significantly promoted breast cancer cell migration, invasion, tube formation in vitro, as well as angiogenesis and metastasis in vivo. In mechanism, the human gene expression profiling was used to determine global transcriptional changes in MDA-MB-231 cells, and we identified autocrine expression of the cytokines CXCL8 and IL11 as the target genes of CUL1 in breast cancer cell migration, invasion, metastasis, and angiogenesis. CUL1 regulated EZH2 expression to promote the production of cytokines, and finally significantly aggravating the breast cancer cell metastasis and angiogenesis through the PI3K–AKT–mTOR signaling pathway. Combined with the previous report about CUL1, we proposed that CUL1 may serve as a promising therapeutic target for breast cancer metastasis.

## Introduction

Breast cancer is the most frequently diagnosed malignancy and the leading cause of cancer-related deaths in women worldwide^1^, and metastasis is responsible for 90% of deaths in breast cancer^2^. Tumor metastasis involves sequentially well-characterized cascades of events, including cell proliferation, migration, invasion, adhesion, and angiogenesis^3^. Recent advances have provided provocative insights regarding the importance of cell-biological and molecular changes on metastasis^4^. Therefore, to uncover the underlying molecular mechanism driving this process might contribute to the perfection of new metastatic paradigms and the development of future therapeutic interventions for metastasis.

Cullin1 (CUL1) is the first and most extensively characterized member of the cullin family. As a well-known scaffolding protein, CUL1 is an essential component of SCF E3 ubiquitin ligase complex. Thus, CUL1 regulates specific ubiquitination of some proteins and then mediates diverse cellular processes, including early embryonic development and cell cycle control^5,6^. Our previous studies have indicated that CUL1 is aberrantly upregulated and significantly correlated with lymphatic and distant metastasis in different types of cancers, such as colorectal cancer^7^, hepatocellular cancer^8^, gastric cancer^9^, and renal cancer cancer^10^. Recently, we have shown that the positive CUL1 staining in cancer tissues predicts poor prognosis of breast cancer patients, and subsequent cancer cell research shows that silencing of CUL1 in cancer cells inhibited the cell migration and invasion abilities through downregulating MMP-2 and MMP-9 expressions^11^. In this study, we conducted a series of in vitro and in vivo experiments and the gene expression profiling to further perfect molecular mechanism of CUL1 in breast cancer metastasis.

## Results

### CUL1 promoted breast cancer migration, invasion, and tube formation in vitro as well as metastasis and angiogenesis in vivo

**Fig. 1:**
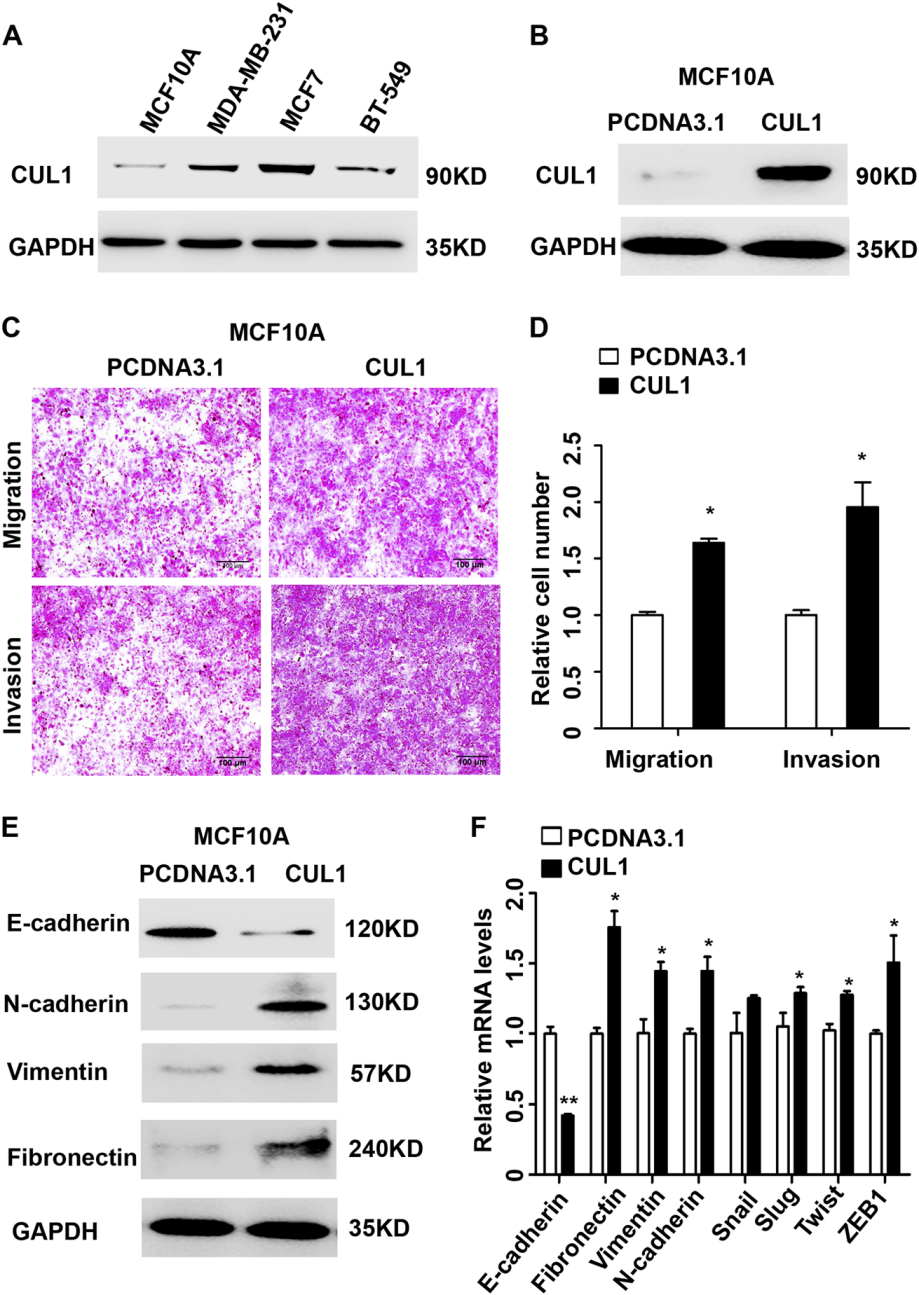
CUL1 induced EMT in MCF10A cells. **a** The CUL1 protein expression in human normal human mammary epithelial cell line MCF10A and breast cancer cell lines were determined by western blotting. **b** MCF10A cells were transiently transfected with CUL1 overexpression (PCDNA3.1-CUL1) and vector control (PCDNA3.1) plasmids. **c** The migration and invasion of MCF10A cells with CUL1 overexpression and vector control. **d** The number of cell migration and invasion per field was counted in five random fields (*n* = 3/group). **e** Western blots of EMT-related markers E-cadherin, β-catenin, N-cadherin, Vimentin, and Fibronectin in MCF10A with CUL1 overexpression and vector control. **f** Relative mRNA expression levels of EMT markers (normalization to GAPDH) in MCF10A cells with CUL1 overexpression and vector control. Data are presented as means ± standard deviations. *P < 0.05, **P < 0.001 (Student’s *t*-test)

To investigate the oncogenic role of CUL1 in breast cancer, we firstly examined the CUL1 expression in the immortalized normal human mammary epithelial cell line MCF10A and a series of breast cancer cell lines including MDA-MB-231, MCF7, BT549 by western blotting. As shown in Fig. 1a, CUL1 expression in the breast cancer cell lines was much higher than the normal mammary epithelial cell line. Then CUL1 was overexpressed in the immortalized normal human mammary epithelial cell line MCF10A cells, and the protein expression of CUL1 was validated (Fig. 1b). We observed that MCF10A cells with CUL1 overexpression significantly increased the migration and invasive abilities than the control ones (Fig. 1c, d), and MCF10A cells with CUL1 overexpression displayed an more elongated fibroblast-like morphology with scattered distribution in culture when compared with the vector control (Figure S1). Usually, the acquisition of stronger migration and invasion capabilities and the change of fibroblast-like morphology of MCF10A cells were associated with epithelial to mesenchymal transition (EMT), so we examined the epithelial and mesenchymal markers. Our results showed that the CUL1 overexpressed MCF10A cells exhibited a dramatically downregulation of E-cadherin; meanwhile the mesenchymal markers N-cadherin, vimentin, and fibronectin were dramatically upregulated (Fig. 1e). Reverse transcriptase polymerase chain reaction (RT-PCR) results also revealed that the mRNA expression change of E-cadherin, fibronectin, vimentin, and N-cadherin in overexpressed MCF10A cells was coincident with the responsive proteins. Moreover, the mRNA levels of well-known EMT inducers, such as Slug, Twist1, and ZEB1 were upregulated in response to CUL1 overexpression (Fig. 1f).

**Fig. 2:**
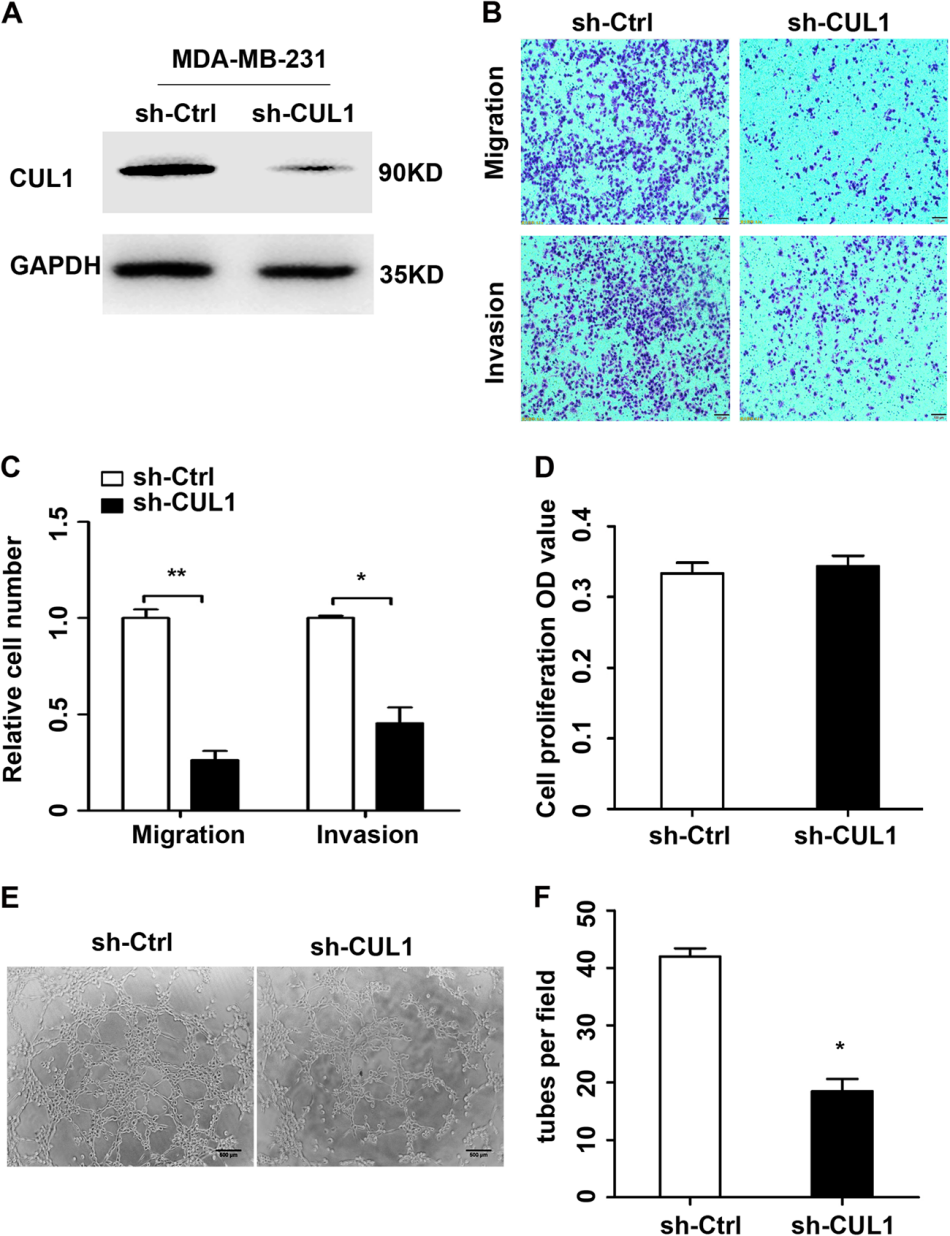
CUL1 knockdown inhibited breast cancer cell migration, invasion, and tube formation in vitro. **a** Lentivirus containing CUL1 knockdown (sh-CUL1) and vector control (sh-Ctrl) plasmids were used to infect MDA-MB-231 cells, respectively. **b** The migration and invasion of MDA-MB-231 cells with CUL1 knockdown and vector control. **c** The number of cell migration and invasion per field was counted in five random fields (*n* = 3/group). **d** The HUVECs cell proliferation absorbance (OD) value in the conditioned medium collected from MDA-MB-231 cells with CUL1 knockdown and vector control. **e** The tube formation by HUVECs in the conditioned medium collected from MDA-MB-231 cells with CUL1 knockdown and vector control. **f** The number of tubes formed per field was counted in five random fields (*n* = 3/group). Data were presented as mean ± SD, *P < 0.05, **P < 0.001 (Student’s *t*-test)

Then we stably infected MDA-MB-231 cells with lentivirus-mediated control shRNA or an effective CUL1 shRNA (Fig. 2a and Figure S2). Since tumor metastasis involves sequential multi-steps, including migration, invasion, angiogenesis, and so on^4^, here we firstly performed the transwell and tube formation assays in vitro. The results of transwell assays demonstrated that CUL1 knockdown in MDA-MB-231 cells significantly inhibited cell migration and invasion by 75% and 55% when compared with respective controls (Fig. 2b, c). For the human umbilical vein endothelial cells (HUVECs) growth and tube formation assays, we firstly collected the 24 h conditioned medium from CUL1 knocked down and vector control MDA-MB-231 cells. The HUVECs growth showed that conditioned medium had little effect on growth of HUVECs compared with the vector control (Fig. 2d). The tube formation assay showed that the conditioned medium from CUL1 knockdown cells significantly decreased the number of complete tubule structures formed by HUVEC cells (Fig. 2e, f).

**Fig. 3:**
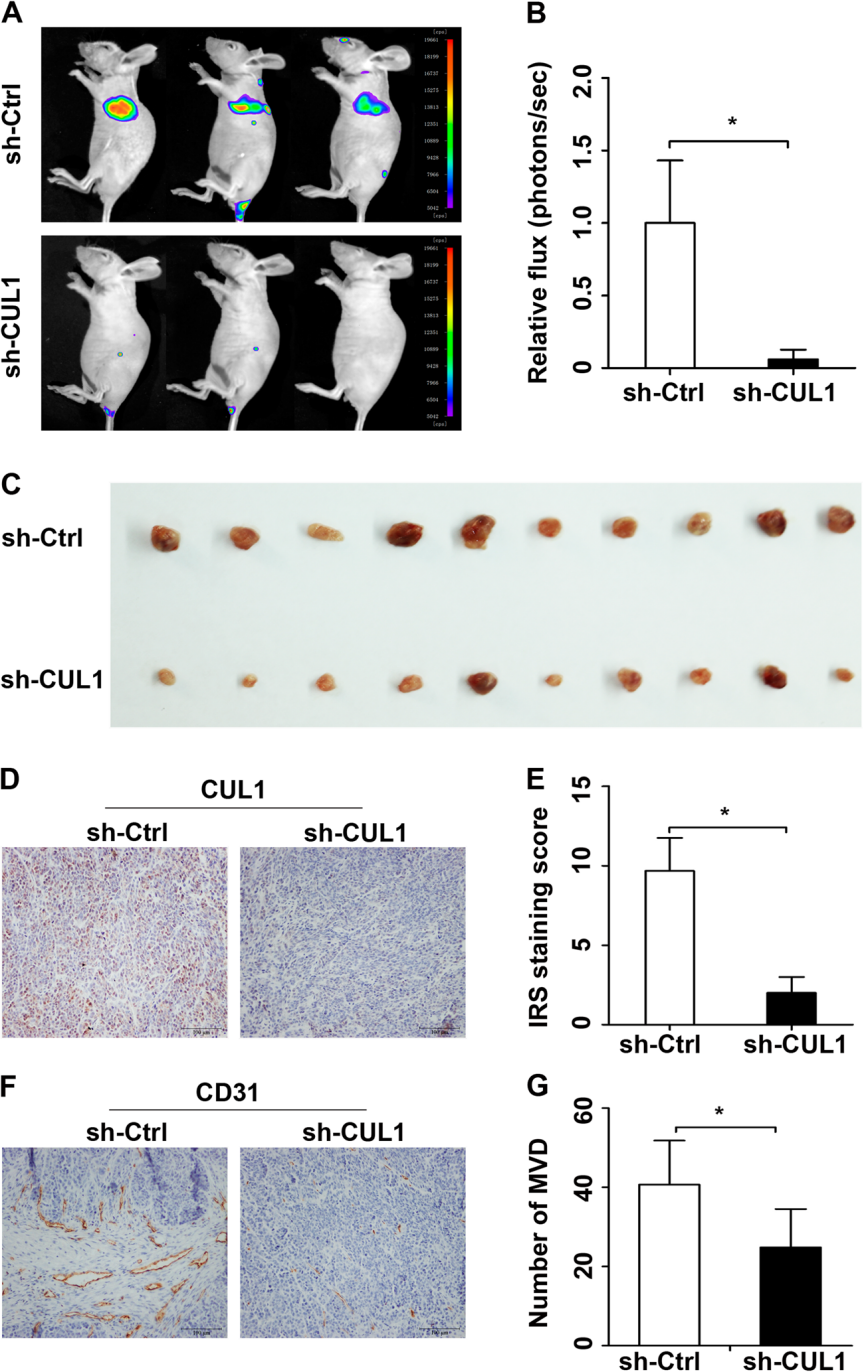
CUL1 knockdown inhibited breast cancer cell metastasis and angiogenesis in vivo. **a** Representative bioluminescence images of mice in the stable CUL1 knockdown group (sh-CUL1) and control group (sh-Ctrl). **b** The fluc activity in sh-CUL1 and sh-Ctrl groups. **c** Photographs of matrigel plugs with sh-CUL1 or sh-Ctrl MDA-MB-231 cells excised from mice after 10 days of growth in vivo. **d** The expression of CUL1 in the matrigel plugs was tested by IHC. **e** The Wilcoxon test was used to evaluate the IRS staining scores of CUL1 (*n* = 3). **f** The expression of CD31 in the matrigel plug was tested by IHC. **g** The number of CD31-positive microvessels were counted (*n* = 3). Data were presented as mean ± SD, *P < 0.05, **P < 0.001 (Student’s *t*-test)

Next, we sought to determine whether CUL1 could promote metastasis and angiogenesis in vivo. In the tail vein metastasis model, the stable CUL1 knockdown and control MDA-MB-231 cells carried luciferase lentivirus were intravenously injected into BALB/C nude mice. After 1 month, bioluminescence imaging was used to monitor the metastatic lesions. Our results exhibited that lung was the main target organ for breast cancer metastasis and the fluc activity in the CUL1 knockdown group is much lower than the control one (Fig. 3a, b). Using the subcutaneous matrigel plug assays, we demonstrated that the reduced vascularization in matrigel plugs containing MDA-MB-231 cells with CUL1 knockdown when compared with the control plugs (Fig. 3c). Accordingly, the immunohistochemical staining demonstrated that CUL1 expression was significantly downregulated in the knockdown group (Fig. 3d, e) and the number of microvessels (CD31 positive) was decreased in the plugs of CUL1 knockdown cells when compared with the controls (Fig. 3f, g).

### Identification of cytokine relative genes as a probable target genes of CUL1 in breast cancer metastasis

**Fig. 4:**
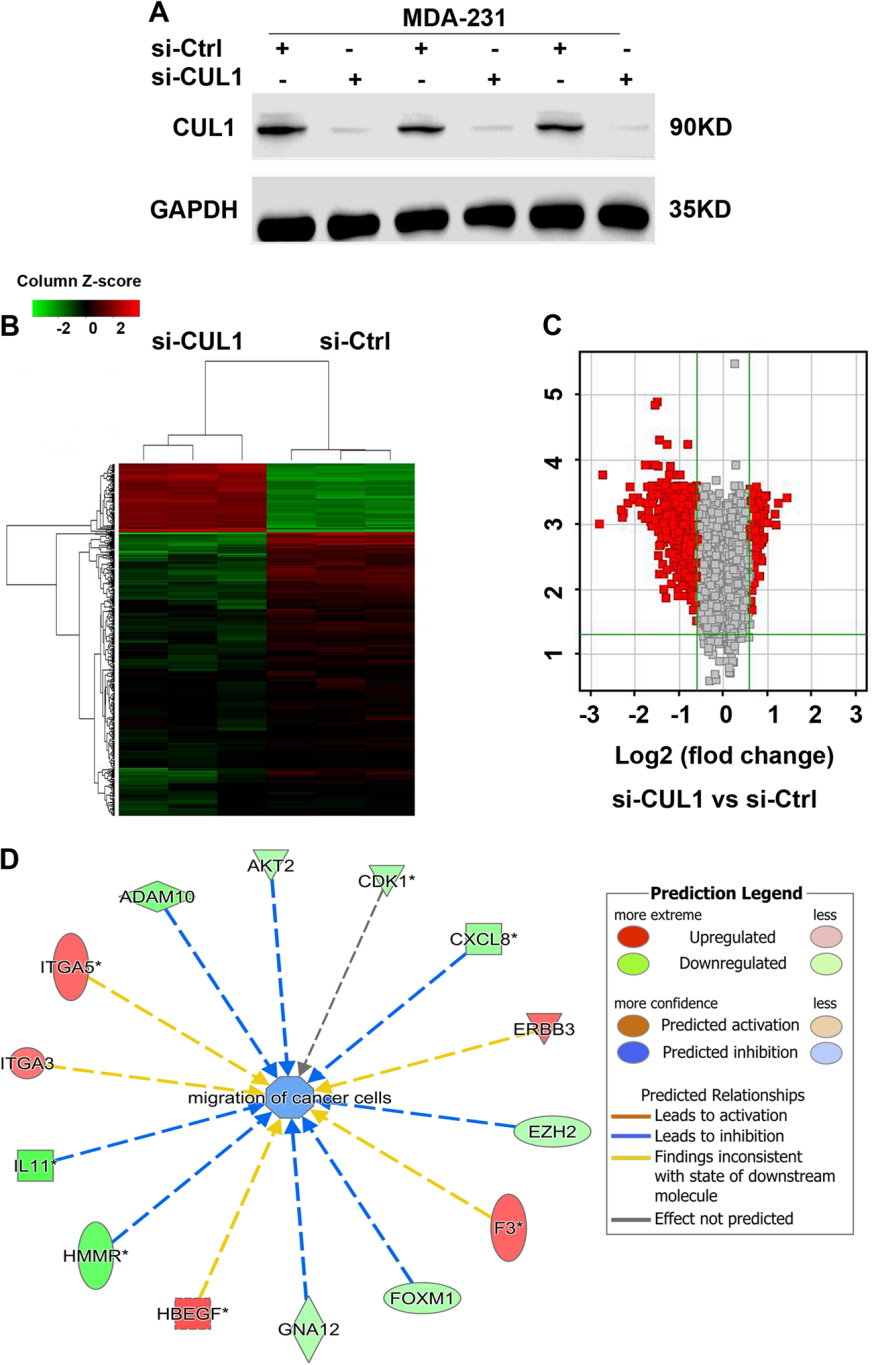
The differential gene expressions were regulated by CUL1. **a** MDA-MB-231 cells were transiently transfected with CUL1 siRNA (si-CUL1) and vector siRNA control (si-Ctrl) in triplicate. **b**, **c** The heat map and volcano plot were used to indicate the significantly differential gene expressions in MDA-MB-231 cells with CUL1 knockdown and vector control. **d** The classification of disease and function was conducted to enrich the differential genes in the migration of cancer cells

**Fig. 5:**
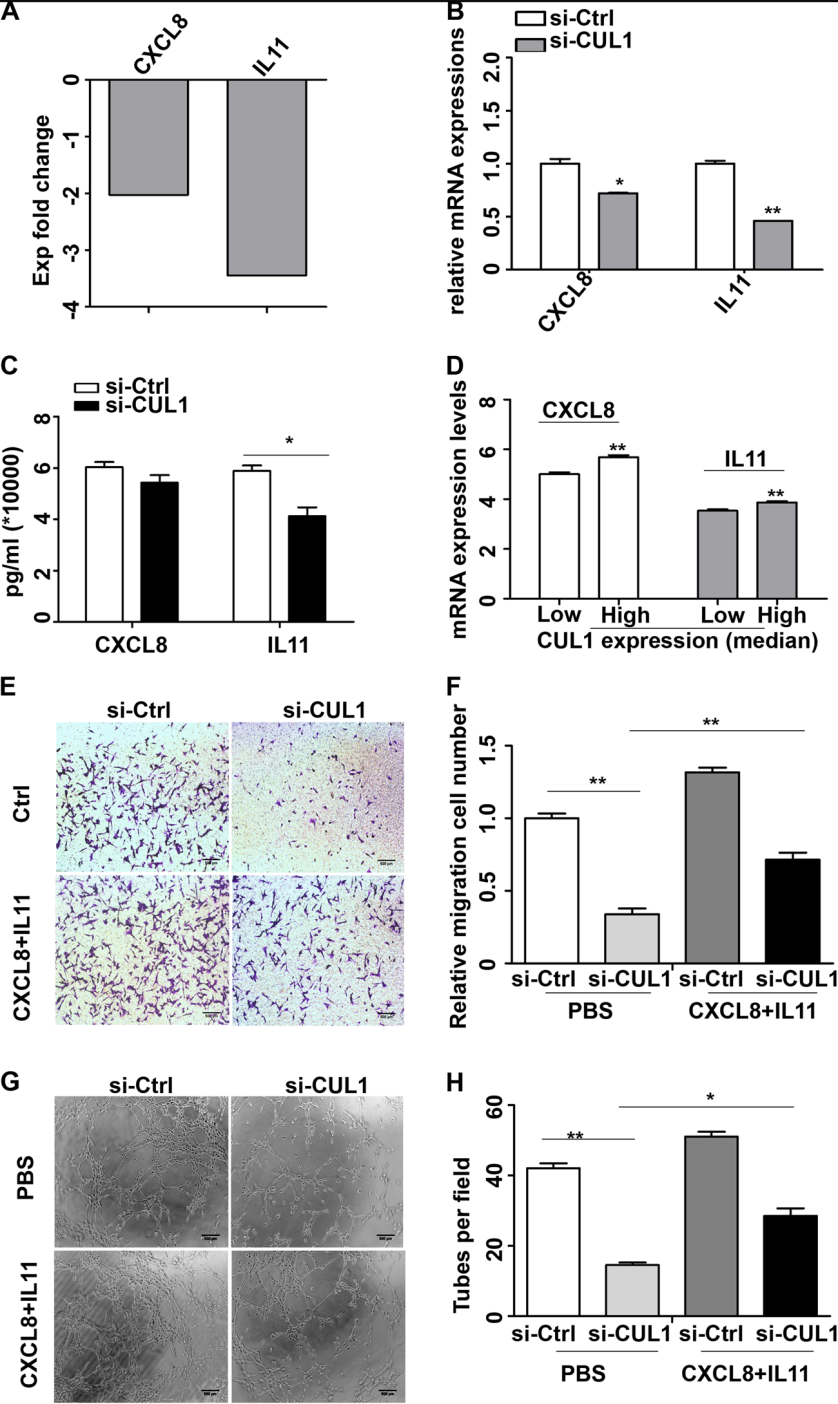
CUL1 knockdown inhibited CXCL8 and IL11 expressions to suppress migration and angiogenesis. **a** Microarray analysis showed the fold changes of CXCL8 and IL11 in the CUL1 knockdown group compared with control groups. **b** Real-time PCR was used to validate the mRNA expressions of CXCL8 and IL11 in CUL1 knockdown and vector control MDA-MB-231 cells. **c** MDA-MB-231 cells were transiently transfected with CUL1 siRNA (si-CUL1) and vector siRNA control (si-Ctrl) for 48 h, and the serum-free medium was added to the cells for 24 h, then the conditioned medium was collected. ELISA analysis was used to test the secreted CXCL8 and IL11 expression levels in the 24 h conditioned medium. **d** The correlation of CUL1 mRNA expressions with CXCL8 and IL11 mRNA expressions in TCGA breast cancer dataset. CUL1 expression was divided into high and low groups based on the average value. **e** MDA-MB-231 cells were seeded in serum-free medium in the upper chamber and the 24 h conditioned medium from CUL1 knockdown and vector control MDA-MB-231 cells containing either supplementary recombinant CXCL8 and IL11 proteins or nothing was placed in the lower chamber, then the representative migration images of MDA-MB-231 cells were showed. **f** The number of cell migration per field was counted in five random fields (*n* = 3/group). **g** The tube formation by HUVECs in the conditioned medium from MDA-MB-231 cells with CUL1 knockdown and vector control which was complemented recombinant CXCL8 and IL11 proteins. **f** The number of tubes formed per field was counted in five random fields (*n* = 3/group) *P < 0.05, **P < 0.001 (Student’s *t*-test)

To further elucidate the mechanism by which CUL1 regulates the breast cancer migration, invasion, metastasis, and angiogenesis, we conducted gene expression profiling of CUL1 knockdown and vector control MDA-MB-231 cells in triplicate (Fig. 4a). The significant differences of expressed genes were identified based on the criteria of fold change >1.5 and *P*-value <0.05. The heat map and volcano plot showed that CUL1 knockdown affected 558 genes (112 upregulated and 446 downregulated) (Fig. 4b, c). The classification of disease and function was conducted to enrich the differential genes. Interestingly, in the function of migration of cancer cells, we found two significantly downregulated cytokine genes CXCL8 and IL11, and the network diagram showed that reduced CXCL8 and IL11 expression inhibited cell migration (Fig. 4d). As cytokines play important roles in tumor migration, invasion, and angiogenesis, the reduction of cytokines was of particular interest^12^. In our microarray data, we found cytokine genes CXCL8 (−2.03-fold) and IL11 (−3.45-fold) were significantly involved in CUL1 knockdown (Fig. 5a). Validation of real-time PCR indicated that CXCL8 and IL11 mRNA levels were significantly inhibited in response to CUL1 knockdown (Fig. 5b). Enzyme-linked immunosorbent assay (ELISA) was used to confirm the alterations of secreted CXCL8 and IL11 proteins. We found that CXCL8 and IL11 expressions in the 24 h conditioned medium from CUL1 knocked down MDA-MB-231 cells were reduced by 6000 and 17,500 pg/ml, respectively, when compared with the medium from the vector control cells (Fig. 5c). Consistently, the significant positive correlations of CUL1 mRNA expressions with CXCL8 and IL11 mRNA expressions were observed in TCGA breast cancer dataset which includes 1153 breast cancer patients (Fig. 5d).

To confirm the role of CXCL8 and IL11 in CUL1 regulated breast cancer cell migration and angiogenesis, we performed CXCL8 and IL11 rescue assays. We firstly collected the 24 h conditioned medium from CUL1 knocked down and vector control MDA-MB-231 cells, then we supplemented 6000 pg/ml recombinant CXCL8 protein and 17,500 pg/ml recombinant IL11 protein into medium. The migration and tube formation assays showed that the inhibited migration ability of MDA-MB-231 cells (Fig. 5e, f) and reduced tubular structure formation of HUVECs (Fig. 5g, h) in conditioned medium from CUL1 knocked down MDA-MB-231 cells were partly rescued by the supplement of recombinant CXCL8 and IL11.

### EZH2 was an important transcriptional factor in CUL1- promoting cytokine gene expressions

**Fig. 6:**
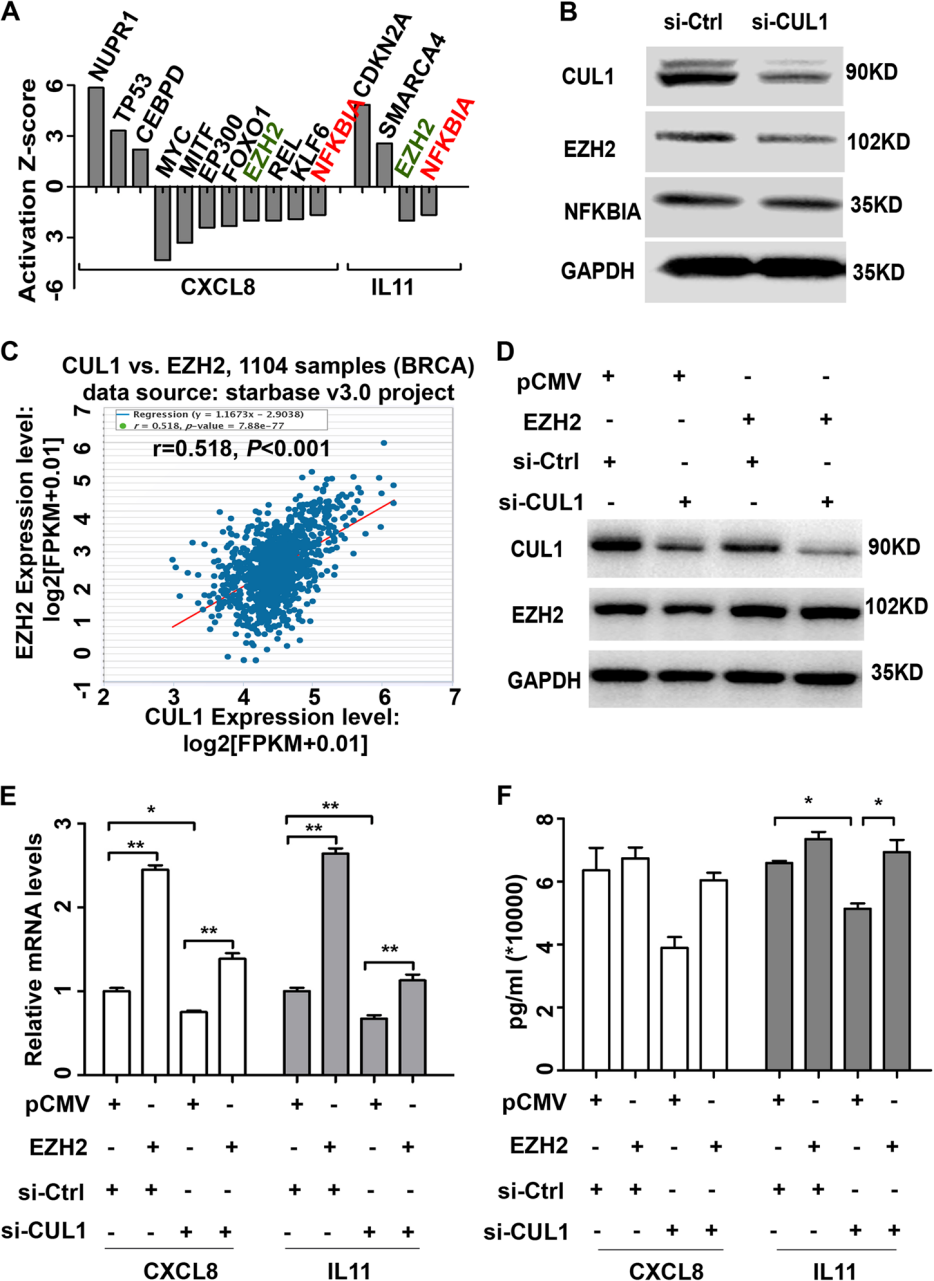
EZH2 was an important transcriptional factor in CUL1 promoting cytokine gene expressions. **a** The activation *Z*-score arithmetic was used to predict the upstream transcriptional regulators of CXCL8 and IL11. **b** Western blotting was used to test the protein expression levels of predicted common upstream regulators ZEH2 and NFKBIA. **c** The correlation of CUL1 mRNA expressions with EZH2 mRNA expressions in 1104 TCGA breast invasive carcinomas from starBase v3.0 project. **d** Western blotting was used to test the expression of CUL1 and EZH2 in the MDA-MB-231 cells transfected with pCMV-EZH2 or vector plasmid, together with either si-CUL1 or si-Ctrl. **e** Real-time PCR was used to explore the CXCL8 and IL11 expressions in the MDA-MB-231 cells transfected with pCMV-EZH2 or vector plasmid, together with either si-CUL1 or si-Ctrl. **f** MDA-MB-231 cells were transiently transfected with pCMV-EZH2 or PCMV vector plasmid, together with either si-CUL1 or si-Ctrl for 48 h, and the serum-free medium was added to the cells for 24 h, then the conditioned medium was collected. ELISA analysis was used to test the protein expression levels of CXCL8 and IL11 in the 24 h conditioned medium. *P < 0.05, **P < 0.001 (Student’s *t*-test)

Based on these findings, we used activation *Z*-score arithmetic to predict the upstream transcriptional regulators of CXCL8 and IL11. Our results showed that several transcriptional factors were predicted to be activated and inhibited (|*Z*-score| ≥ 1.5) (Fig. 6a). Western blotting was used to test the protein expression levels of predicted common upstream regulators ZEH2 and NFKBIA, and the results showed that EZH2 protein expression was significantly inhibited by CUL1 knockdown (Fig. 6b). Simultaneously, to validate the relationship of CUL1 with EZH2, we used the starBase v3.0 project (http://starbase.sysu.edu.cn/panCancer.php) including the gene expression data of 1104 TCGA breast invasive carcinomas and found CUL1 expression levels were significantly positively correlated with EZH2 expressions (*r* = 0.518, *P* < 0.001; Fig. 6c).

**Fig. 7:**
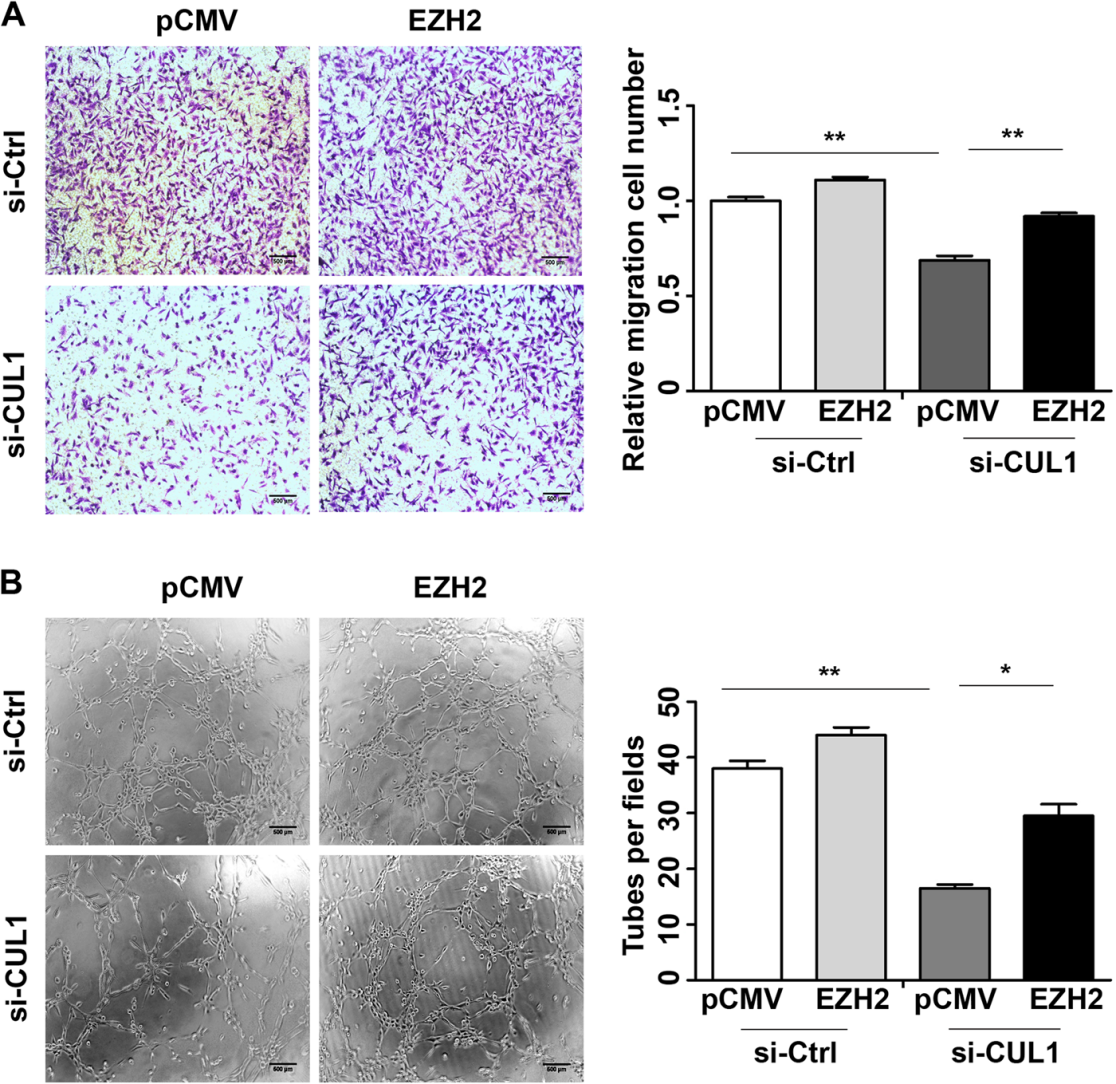
CUL1 exerted its effect on breast cancer cells metastasis through EZH2. **a** The migration of MDA-MB-231 cells transiently transfected with pCMV-EZH2 or PCMV vector plasmid together with either si-CUL1 or si-Ctrl. **b** The number of cell migration and invasion per field was counted in five random fields (*n* = 3/group). **c** The tube formation by HUVECs in the 24 h conditioned medium collected from MDA-MB-231 cells transfected with pCMV-EZH2 or PCMV vector plasmid together with either si-CUL1 or si-Ctrl. **f** The number of tubes formed per field was counted in five random fields (*n* = 3/group). *P < 0.05, **P < 0.001 (Student’s *t*-test)

Then we investigated whether CUL1 exerted its effect on breast cancer cells metastasis through EZH2. We overexpressed pCMV-EZH2 or pCMV vector plasmids in CUL1 knockdown or control MDA-MB-231 cells (Fig. 6d), and collected their mRNA and 24 h conditioned medium. The real-time PCR and ELISA assays showed that EZH2 overexpression rescued CXCL8 and IL11 mRNA and protein production in CUL1 knockdown groups (Fig. 6e, f). Some studies have reported that nuclear factor-κB (NF-κB) activation is critical for cytokines expression and dependent upon high EZH2 expression in triple-negative breast cancer cells^13,14^. In order to test whether NF-κB signal pathway plays an important role in EZH2-regulated cytokines expression, we performed luciferase reporter gene assay. In accordance with the previous findings^13,14^, our results showed that EZH2 overexpression rescued CUL1 knockdown-decreased the transcriptional activity of NF-κB (Figure S3). Moreover, cell migration and tube formation assays also showed that EZH2 overexpression rescued CUL1 knockdown-reduced cell migration (Fig. 7a, b) and tubular structure formation (Fig. 7c, d).

### CUL1 inducing cytokines activated PI3K–AKT signaling pathway

**Fig. 8:**
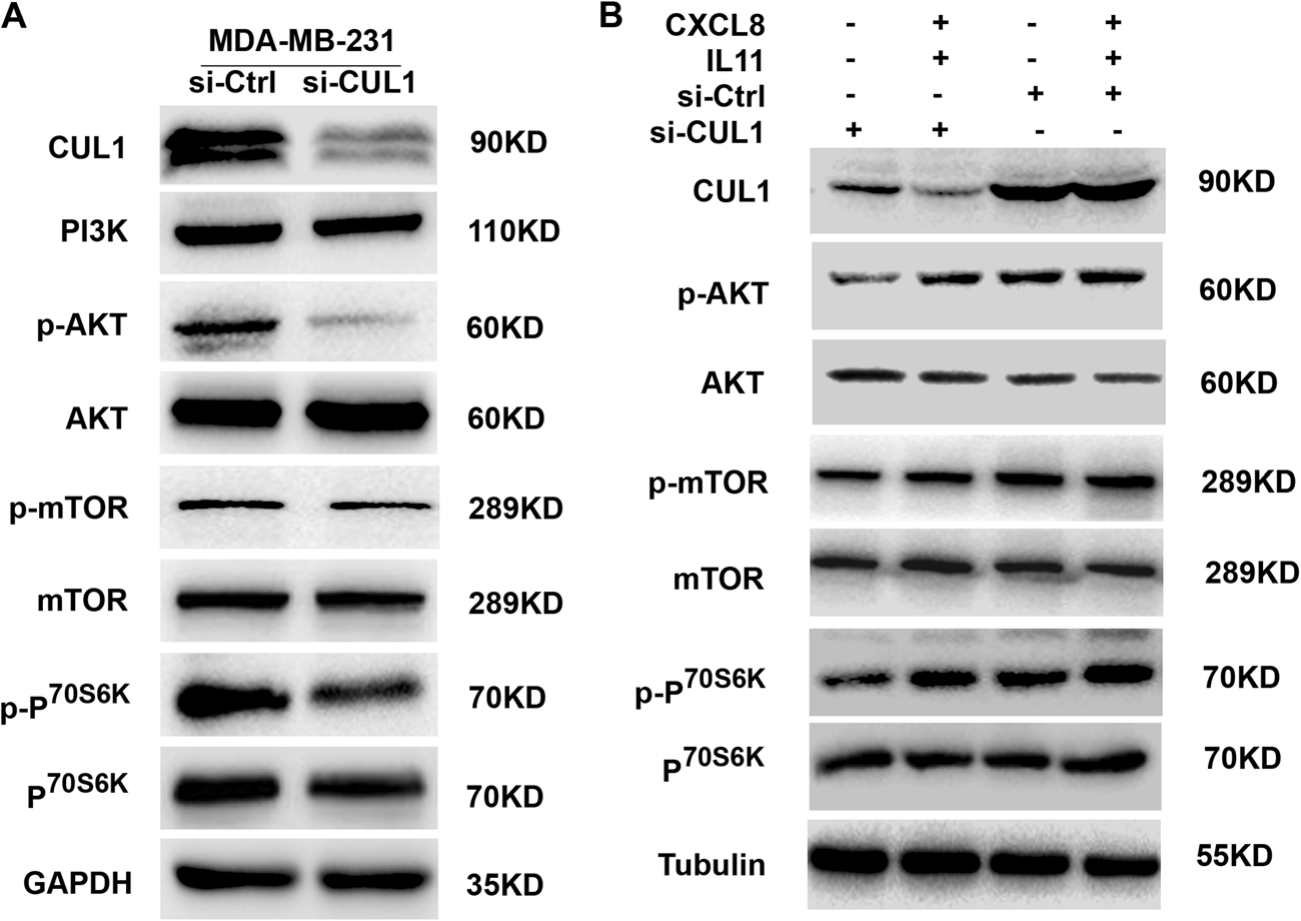
CUL1 inducing cytokines activated PI3K–AKT signaling pathway. **a** The expressions of PI3K–AKT–mTOR signaling pathway-related proteins, such as PI3K, p-AKT, AKT, p-mTOR, mTOR, p-P^70S6K^, and P^70S6K^ in MDA-MB-231 cells with CUL1 knockdown or vector control. **b** MDA-MB-231 cells were transiently transfected with si-CUL1 and si-Ctrl for 48 h and these cells were continued culturing in serum-free medium with or without supplementary recombinant CXCL8 and IL11 proteins for 24 h, then western blotting was used to test the expressions of p-AKT, AKT, p-mTOR, mTOR, p-P^70S6K^, and P^70S6K^

Since PI3K–AKT–mTOR signaling is one of the critical downstream pathways of CXCL8 and IL11 in mediating cancer metastasis and EMT^15,16^, to test whether PI3K–ATK–mTOR signaling pathway involved in CUL1 induced tumor metastasis, we knocked down CUL1 in MDA-MB-231 cells, and western blotting results showed that CUL1 knockdown significantly suppressed p-AKT, p-mTOR, and p-P^70S6K^ protein levels (Fig. 8a). Then we treated CUL1 knocked down or vector control MDA-MB-231 cells with human recombinant CXCL8 (6000 pg/ml) and IL11 (17500 pg/ml) proteins. As shown in Fig. 8b, p-AKT, p-mTOR, and p-P^70S6K^ levels which were suppressed by CUL1 knockdown were subsequently recovered by treatment with cytokines. Collectively, CUL1 inducing CXCL8 and IL11 can exhibit a pronounced activated effect on downstream PI3K–AKT–mTOR signaling.

## Discussion

Our previous study has indicated that CUL1 is positively associated with poor 5-year overall and disease-specific survival of breast cancer patients^11^. Most of breast cancer-related deaths are due to metastasis diseases. Since tumor metastasis involves sequential multi-steps, including angiogenesis, migration, invasion, and so on^4^, in our study, we designed a series of experiments to explore the roles of CUL1 in breast cancer metastasis. Our data showed that CUL1 was highly expressed in breast cancer cells when compared with normal mammary epithelial cells. Overexpression of CUL1 in normal mammary epithelial cells significantly increased the cell migration and invasion and showed the elongated fibroblast-like morphology through inducing EMT, while knockdown of CUL1 inhibited breast cancer cell migration, invasion, and tube formation in vitro. Using intravenous tumor metastasis model and angiogenesis in vivo, we indicated that CUL1 knockdown significantly reduced the lung metastasis and angiogenesis. Together, these results showed that CUL1 played as an important regulator of breast cancer metastasis.

With regard to the potential of CUL1 in breast cancer migration, invasion, angiogenesis, and metastasis, we further investigated the molecular mechanism on the functional properties. In the study, microarray analysis was used to determine global transcriptional changes in cell lines and revealed that hundreds of genes were regulated by CUL1 knockdown. The analyses of disease and function network showed cytokines were significantly enriched in the migration function of cancer cells. Our data revealed that both mRNA and protein of cytokines CXCL8 and IL11 were regulated by CUL1. It is well known that autocrine cytokines regulate divergent tumor cell behaviors and tumor microenvironment^17^. Autocrine cytokines CXCL8 and IL11 induce neo-angiogenesis through activation of the vascular endothelial growth factor pathway. Additionally, they enhance the activity of MMP-2 and MMP-9 which in turn increase the metastatic activity of the underlying malignancy^17–20^. These data are accorded with our previous researches in breast cancer and renal cell carcinoma that CUL1 increases cancer cell metastasis through upregulating the activity of MMP-2 and MMP-9 (refs. ^10,11^). Moreover, using the clinical samples, we found that CUL1 mRNA expression positively correlated with CXCL8 and IL11 mRNA levels. The roles of CXCL8 and IL11 in CUL1-regulated breast cancer metastasis were further validated by CXCL8 and IL11 rescue assays. Though here CUL1 knockdown did not significantly reduced the expression of CXCL8, other researches have indicated that critical inflammatory genes, especially the coordination of autocrine expressions of the pro-inflammatory cytokines IL6 and CXCL8, play important roles in the growth of triple-negative breast cancer cells^13^. Moreover, IL11, along with IL6, which belongs to the members of the glycoprotein (GP)130 (GP130) cytokine family, exclusively utilizes GP130 homodimers^20^. In our study, we tested the joint effect of CXCL8 and IL11 in the rescue assays. Our data indicated that CXCL8 and IL11 contributed to CUL1-regulated breast cancer metastasis.

Having seen cytokines changes resulted from the CUL1 knockdown, here we went on elucidating the signaling pathways how CUL1 regulated these two cytokines. Using the *Z*-score arithmetic, several transcriptional factors were predicted to be activated and inhibited, and subsequent protein results reminded us that EZH2 might play as the most important common transcriptional regulator. EZH2 is known as the catalytic subunit of the polycomb repressive complex 2, which is a highly conserved histone methyltransferase that methylates lysine 27 of histone 3 (ref. ^21^). Studies have indicated that EZH2 can act as a gene silencer or a transcriptional gene activator. Recently, researchers have found that independence of its methyltransferase activity, EZH2 activates NF-κB target genes in breast cancer^14^, and EZH2 is required for the cytokine production^13^. Using TCGA breast cancer dataset, we found that CUL1 mRNA levels positively correlated with EZH2 mRNA expression. The role of EZH2 in CUL1-regulated cytokine expression of CXCL8 and IL11 was confirmed by EZH2 overexpression in CUL1 knockdown cells. Moreover, we showed that EZH2 overexpression rescued CUL1 knockdown-decreased transcriptional activities of NF-κB, which is accordant with previous findings that NF-κB activation is critical for cytokines expression and dependent upon high EZH2 expression in triple-negative breast cancer cells^13,14^. At the same time, we found that EZH2 overexpression blocked CUL1-reduced CXCL8 and IL11 expression and subsequent cell migration and tube formation. These findings suggested that CUL1 could stimulate the production of cytokines through inducing EZH2 expression to regulate breast cancer metastasis.

Considering the PI3K–AKT–mTOR pathway plays a central role in tumor progression by transmitting signal transduction events in response to extracellular stimuli, for example, PI3K can receive signals from growth factor receptors, integrins, and cytokine receptors^22^, and there are evidences suggesting that the PI3K–AKT–mTOR pathway associated with CXCL8 and IL11 signaling may contribute to cancer cell progression and metastasis, and using the PI3K inhibitor can directly suppresses the invasion activity induced by CXCL8 and IL11 (refs. ^20,23^). In this study, we elucidated this signaling pathway on CUL1-regulated breast cancer metastasis. We found that CUL1 inhibited the activation of PI3K–AKT–mTOR signaling pathway and supplementary CXCL8 and IL11 blocked CUL1 knockdown inhibiting PI3K–AKT–mTOR activation. Therefore, our data suggested that the activation of PI3K and its downstream effector AKT by CUL1-induced CXCL8 and IL11 may play a role in cancer metastasis.

## Conclusion

Taken together, our data highlighted that CUL1 regulated EZH2 expression to promote the production of autocrine expression of the autocrine cytokines CXCL8 and IL11, and finally significantly aggravating the breast cancer cell metastasis through the PI3K–AKT–mTOR signaling pathway. Combined with our previous report about CUL1, we proposed that CUL1 may serve as a promising therapeutic target for breast cancer metastasis.

## Materials and methods

### Cell lines and animals

Human MCF10A, MCF7, MDA-MB-231 cell lines were obtained from ATCC, BT549 and HUVECs were purchased from the Shanghai Institute of Biochemistry and Cell Biology, Chinese Academy of Sciences (Shanghai, China). MCF10A cells were cultured as previously described^24^. MCF7 and BT549 cells were cultured in RPMI-1640 medium supplemented with 10% fetal bovine serum (FBS). MDA-MB-231 cells were cultured in L15 medium with 10% FBS. The cells were grown at 37 °C in the presence of 5% CO_2_ in a humidified incubator. Female BALB/c nude mice, 4–6 weeks old, were purchased from NLARSH China (Shanghai, China), and maintained under specific pathogen-free conditions. The experiments in vivo were approved by the Animal Care Committee of Xuzhou Medical University.

### Plasmids, siRNA, transient transfections, and virus infection

PCDNA3.1-CUL1, pCMVHA-EZH2 plasmids (Addgene), PCDNA3.1-vector and pCMV vector were confirmed before using by DNA sequencing. Nonspecific control siRNA (Qiagen) and CUL1 siRNA (Dharmacon) were purchased^11^. The plasmids and siRNAs were transiently transfected into cells using Lipofectamine 2000 transfection reagent (Invitrogen, Shanghai, China) following the manufacturer’s protocol. The pEGFP-C1-Sh-CUL1 (sh-CUL1) and pEGFP-C1-Sh-control (sh-Ctrl) (GenePharma) were generated and confirmed before using by DNA sequencing, and lentivirus was used to pack these two plasmids and infected the MDA-MB-231 cells following the manufacturer’s protocol. The cells were stably selected with 2 μg/ml puromycin for 2 weeks.

### Western blotting and antibodies

Western blotting was carried out as previously reported^25^. The anti-CUL1 (1:5000; Abcam, USA), anti-E-cadherin (1:2000; BD Biosciences, USA), anti-β-catenin (1:2000; BD Biosciences, USA), anti-N-cadherin (1:2000; BD Biosciences, USA), anti-Vimentin (1:2000; BD Biosciences, USA), anti-Fibronectin (1:2000; BD Biosciences, USA), anti-EZH2 (1:1000; Proteintech, China), anti-NFKBIA (1:1000; Abcam, USA), anti-PI3K (1:1000; CST, USA), anti-AKT (1:1000; CST, USA), anti-pAKT (1:1000; CST, USA), anti-mTOR (1:1000; CST, USA), anti-p-mTOR (1:1000; CST, USA), anti-P^70S6K^ (1:1000; CST, USA), and anti-p-P^70S6K^ (1:1000; CST, USA) were used for primary antibody incubation at 4 °C overnight. The anti-GAPDH (1:1000; Beyotime Biotechnology, China) and anti-tubulin were used for the protein loading control. Each blot was repeated three times.

### RNA extraction, RT-PCR, and real-time PCR analysis

TRIzol reagent (Invitrogen) was used to extract RNA from cells, and the cDNA was generated with HiScript II 1st Strand cDNA Synthesis Kit (Vazyme Biotech). Real-time PCR was carried out on ABI-7500 using SYBR Green Real-time PCR Master Mix (Vazyme Biotech). GAPDH was used for normalization of real-time PCR data. Primer sequences are listed below.

**Table:** 

**Primer names**	**Primer sequences**
GAPDH-For	5′-AAGGTCGGAGTCAACGGATTTG-3′
GAPDH-Rev	5′-CCATGGGTGGAATCATATTGGAA-3′
CUL1-For	5′-GCCGTGTCGCACGCAG -3′
CUL1-Rev	5′-GTCCCGCCAAGGTCTAGCTG -3′
E-cadherin-For	5′-GACAACAAGCCCGAATT-3′
E-cadherin -Rev	5′-GGAAACTCTCTCGGTCCA-3′
Fibronectin-For	5′-CAGTGGGAGACCTCGAGAAG-3′
Fibronectin-Rev	5′-TCCCTCGGAACATCAGAAAC-3′
Vimentin-For	5′-GAGAACTTTGCCGTTGAAGC-3′
Vimentin-Rev	5′-GCTTCCTGTAGGTGGCAATC-3′
N-cadherin-For	5′-CGGGTAATCCTCCCAAATCA-3′
N-cadherin-Rev	5′-CTTTATCCCGGCGTTTCATC-3′
Snail-For	5′-GCAAATACTGCAACAAGG-3′
Snail-Rev	5′-GCACTGGTACTTCTTGACA-3′
Slug-For	5′-AGATGCATATTCGGACCCAC -3′
Slug-Rev	5′-CCTCATGTTTGTGCAGGAGA-3′
Twist-For	5′-GGAGTCCGCAGTCTTACGAG-3′
Twist-Rev	5′-TCTGGAGGACCTGGTAGAGG-3′
ZEB1-For	5′-TGCACTGAGTGTGGAAAAGC-3′
ZEB1-Rev	5′-TGGTGATGCTGAAAGAGACG-3′
CXCL8-For	5′-CCACCGGAGCACTCCATAAG-3′
CXCL8-Rev	5′-GATGGTTCCTTCCGGTGGTT-3′
IL11-For	5′-ACATGAACTGTGTTTGCCGC-3′
IL11-Rev	5′-AGCTGGGAATTTGTCCCTCAG-3′

### Cell migration and invasion assay

The migration and invasion assays were performed as described before^24^. In brief, the transwell filter inserts with a pore size of 8 μm were coated without or with matrigel for the cell migration and invasion assays, respectively. 1 × 10^5^ cells (for migration) and 2 × 10^5^ cells (for invasion) were respectively seeded in serum-free medium in the upper chamber. After 24 h incubation at 37 °C, cells in the upper chamber were carefully removed with a cotton swab and the cells that had traversed the membrane were fixed in methanol, stained with Crystal violet (0.04% in water; 100 μl), and counted the permeating cells under the inverted microscope and photographed.

### HUVECs growth and tube formation assays

1 × 10^6^ stable CUL1 knockdown and control MDA-MB-231 cells were cultured in 60-mm plates with 2 ml fresh serum-free medium for 24 h, and then conditioned medium was collected. For HUVEC growth assay, the endothelial cells were seeded in a 96-well plate at 5 × 10^3^ cells/well in 0.1 ml conditioned medium for 24 h, and then CCK-8 assay was performed following the manufacturer’s protocol (Dojindo). For the tube formation, a 48-well plate was coated with 200 μl matrigel™ (BD Biosciences) and kept at 37 °C for 0.5 h. Then, 1.5 × 10^4^ HUVECs were suspended in 200 μl of conditioned medium and applied to the precoated 48-well plate. After incubation at 37 °C for another 8 h, photos were taken under a microscope, and the complete tubular structures were counted.

### Tumor metastasis model, angiogenesis in vivo

To produce metastasis model in vivo, the BALB/c nude mice were randomly divided into two groups. 1 × 10^6^ luciferase lentivirus-infected stable CUL1 knockdown and control MDA-MB-231 cells were suspended in 100 μl phosphate-buffered saline (PBS) and injected intravenously through tail vein, respectively. For angiogenesis in vivo, the BALB/c nude mice were randomly divided into two groups, and 1 × 10^7^ lentivirus-infected stable CUL1 knockdown and control MDA-MB-231 cells were suspended in 100 μl PBS, mixed with 200 μl matrigel and subcutaneously implanted into the flanks of nude mice. Animal experiments were performed as previously described^25,26^.

### Immunohistochemistry (IHC) and assessment of IHC

The excised plugs from the angiogenesis in vivo were fixed in 10% buffered formalin, and then these paraffin-embedded tissues were applied in further sectioning and staining for CUL1 and CD31 as described previously^26^. Finally, the intensity of CUL1 staining was scored and the number of CD31- positive cells in five different fields was counted as reported before^25^.

### Gene expression profiling and network analysis

Human gene expression microarray (Affymetrix) was used to analyze gene expressions collected from MDA-MB-231 cells with transiently transfected by CUL1 siRNA or control (Genechem, Shanghai, China). The Ingenuity Pathway Analysis software was used to analyze the classification of disease and function and the network of upstream transcriptional regulators.

### Public domain data

In order to identify the clinical correlation of CUL1 with CXCL8 and IL11 in breast cancer, we downloaded the clinical data from the TCGA database (http://cancergenome.nih.gov) and the corresponding RNA-sep data from the UCSC Xena (https://xenabrowser.net/datapages/). Moreover, we used the starBase v3.0 project (http://starbase.sysu.edu.cn/panCancer.php) to analyze the correlation of CUL1 gene expression with EZH2 expression.

### ELISA analysis and human recombinant proteins

Secreted CXCL8 and IL11 protein in the 24 h conditioned medium were measured by the human CXCL8 and IL11 ELISA kits from Elabscience Biotechnology according to the manufacturer’s instructions. Human recombinant CXCL8 (Proteintech Group) and IL11 (R&D systems) were reconstituted to 0.25 μg/μl using sterile water and 50 μg/ml using sterile PBS.

### Luciferase reporter gene assay

Cells were seeded onto 24-well plates (5 × 10^*4^cells/well), and co-transfected with 0.5 μg pGL6-pNF-κB-luc luciferase reporter plasmid (Beyotime), 10 ng pRLSV40, and si-CUL1/PCMV or si-Ctrl/PCMV or si-CUL1/EZH2 or si-Ctrl/EZH2 for 24 h, and then cell lysates were prepared according to Promega’s instruction manual. Luciferase activity was measured with a dual-Luciferase Reporter Assay System (Promega, USA) and the activity was normalized against the Renilla luciferase gene.

### Statistical analysis

All the statistical analyses were performed by STATA statistical software (version 15; StataCorp, College Station, TX). The Wilcoxon test (raw CUL1 IHC scores) and Student’s *t*-test were used to evaluate the significance, a *P*-value of <0.05 was deemed statistically significant, and all tests were two-sided.

## Supplementary information

Figure S1

Figure S2

Figure S3

Supplementary figure legends
