# Supplementary figures and images for: CUL1 promotes breast cancer metastasis through regulating EZH2-induced the autocrine expression of the cytokines CXCL8 and IL11
Source: Cell Death Dis. 2018 Dec 18;10(1):2. doi: 10.1038/s41419-018-1258-6 (PMC6315038; doi:10.1038/s41419-018-1258-6)

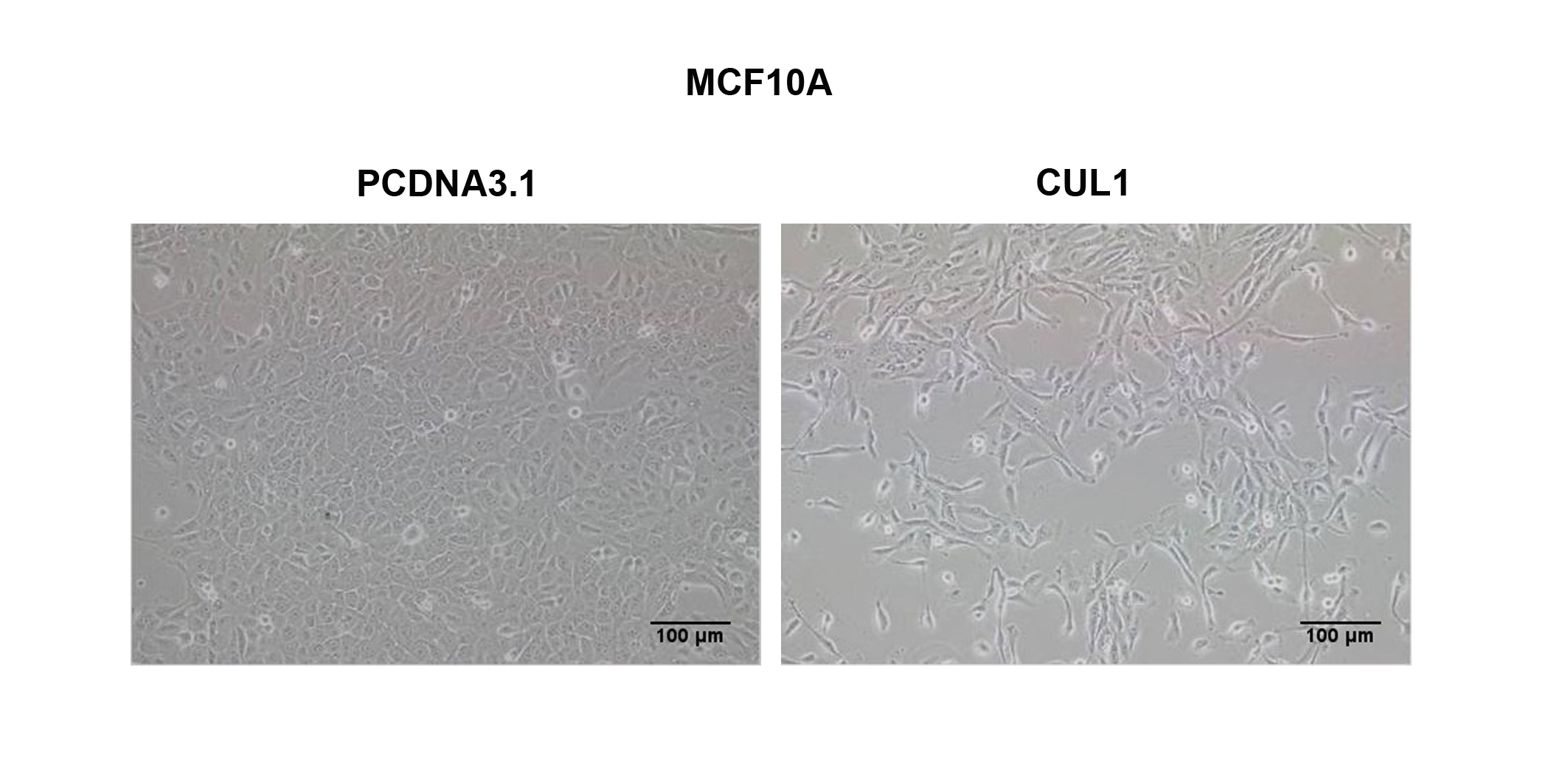

Supplement: Supplementary file 1 — Figure S1 [file 41419_2018_1258_MOESM1_ESM.tif]

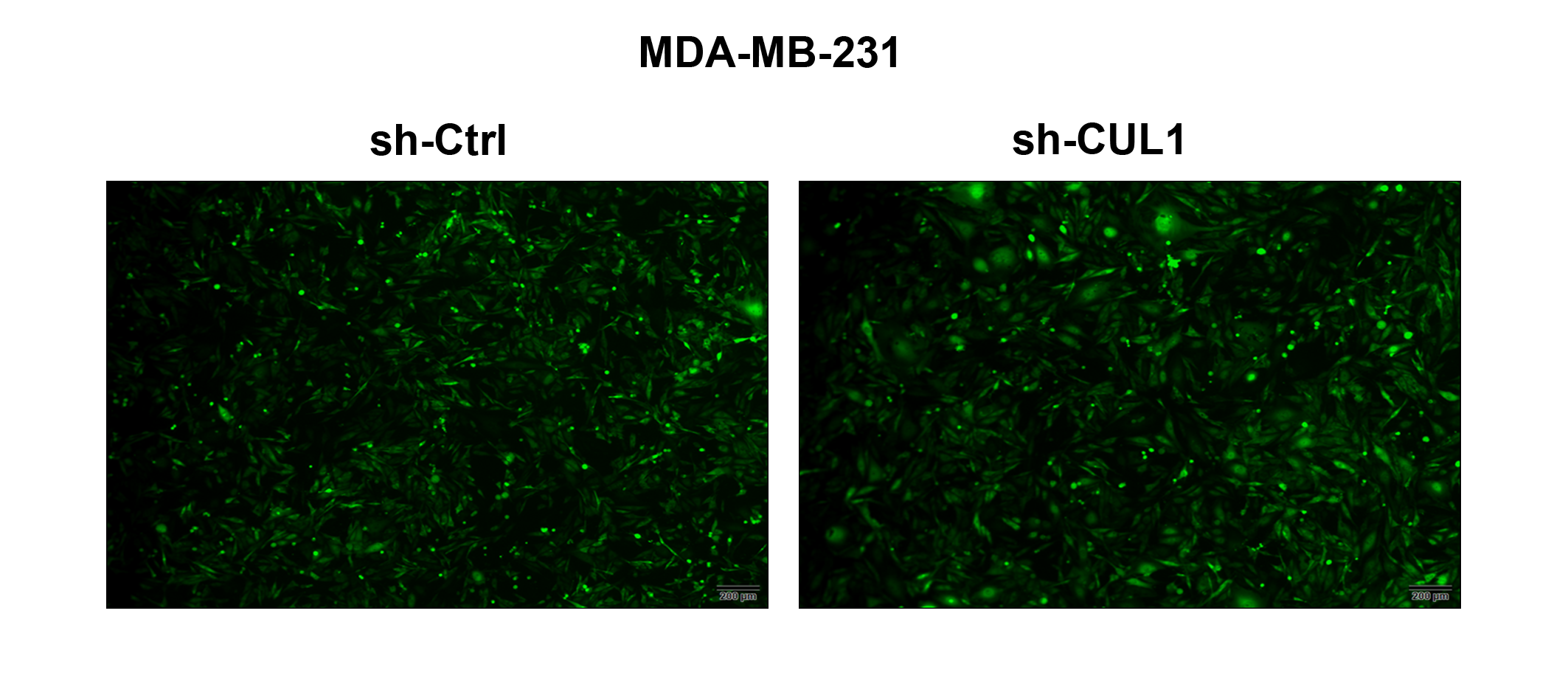

Supplement: Supplementary file 2 — Figure S2 [file 41419_2018_1258_MOESM2_ESM.tif]

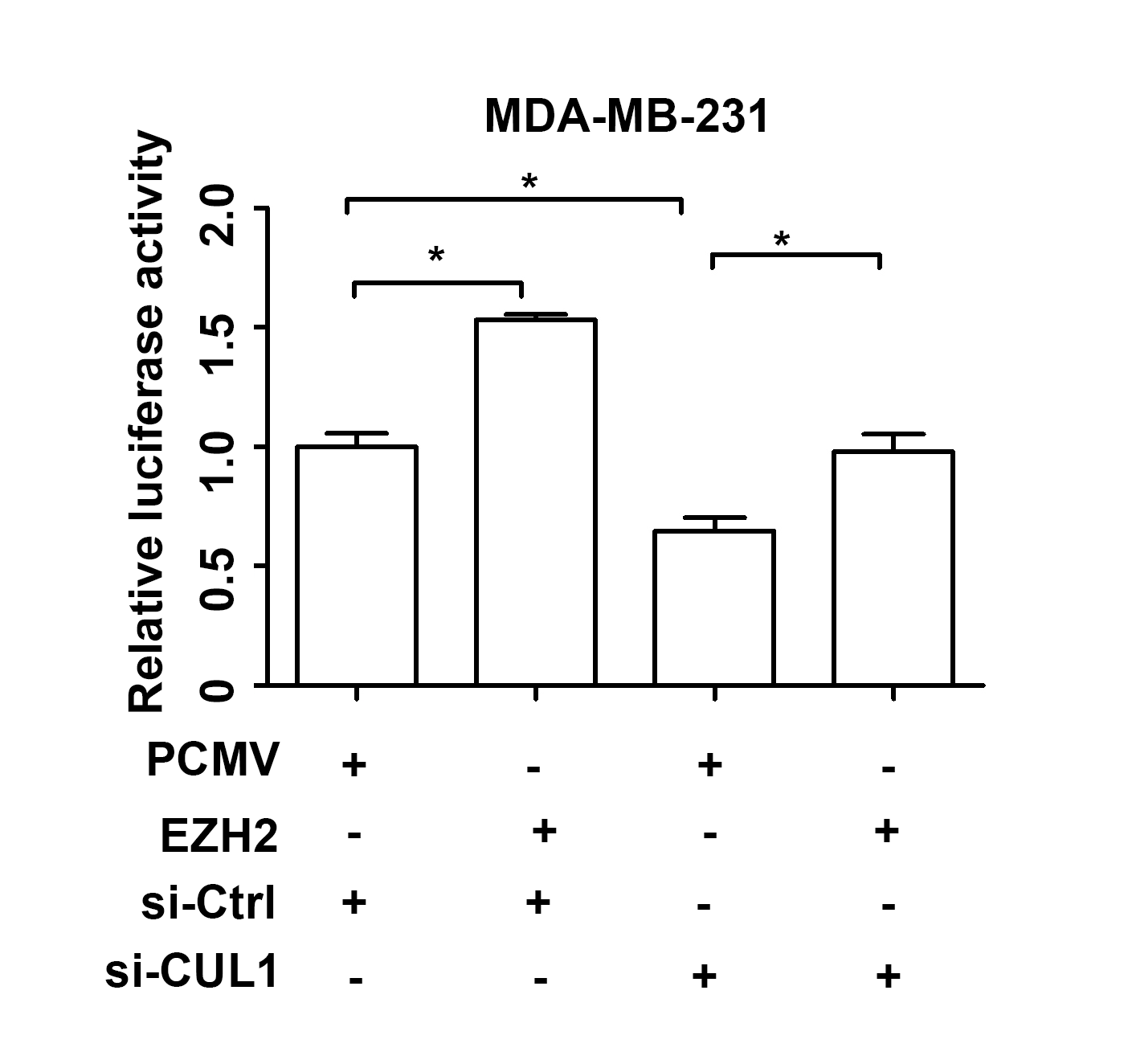

Supplement: Supplementary file 3 — Figure S3 [file 41419_2018_1258_MOESM3_ESM.tif]
