# Supplementary material for: CUL1 promotes breast cancer metastasis through regulating EZH2-induced the autocrine expression of the cytokines CXCL8 and IL11
Source: Cell Death Dis. 2018 Dec 18;10(1):2. doi: 10.1038/s41419-018-1258-6 (PMC6315038; doi:10.1038/s41419-018-1258-6)
Supplement: Supplementary file 4 — Supplementary figure legends [file 41419_2018_1258_MOESM4_ESM.docx]

**Figure S1** The morphologic change of MCF10A cells with CUL1 overexpression or vector control.

**Figure S2** The green fluorescent images of MDA-MB-231 cells with lentivirus-mediated control shRNA (sh-Ctrl) or CUL1 shRNA (sh-CUL1).

**Figure S3** The luciferase reporter gene assay tested the NF-κB activity in MDA-MB-231 cells transfected with pCMV-EZH2 or PCMV vector plasmid, together with either si-CUL1 or si-Ctrl. ^*^*P* < 0.05 (Student’s t-test).
